# Influence of macromolecule baseline on ^1^H MR spectroscopic imaging reproducibility

**DOI:** 10.1002/mrm.26103

**Published:** 2016-01-22

**Authors:** Rebecca Birch, Andrew C. Peet, Hamid Dehghani, Martin Wilson

**Affiliations:** ^1^ PSIBS Doctoral Training CentreUniversity of BirminghamUnited Kingdom; ^2^ Birmingham University Imaging Centre (BUIC), School of PsychologyUniversity of BirminghamUnited Kingdom; ^3^ Department of OncologyBirmingham Children's Hospital NHS Foundation TrustBirminghamUnited Kingdom; ^4^ School of Cancer SciencesUniversity of BirminghamUnited Kingdom; ^5^ School of Computer ScienceUniversity of BirminghamKingdom.

**Keywords:** macromolecule, MRSI, reproducibility, TE, baseline

## Abstract

**Purpose:**

Poorly characterized macromolecular (MM) and baseline artefacts are known to reduce metabolite quantitation accuracy in ^1^H MR spectroscopic imaging (MRSI). Increasing echo time (TE) and improvements in MM analysis schemes have both been proposed as strategies to improve metabolite measurement reliability. In this study, the influence of TE and two MM analysis schemes on MRSI reproducibility are investigated.

**Methods:**

An experimentally acquired baseline was collected using an inversion recovery sequence (TI = 750 ms) and incorporated into the analysis method. Intrasubject reproducibility of MRSI scans, acquired at 3 Tesla, was assessed using metabolite coefficients of variance (COVs) for both experimentally acquired and simulated MM analysis schemes. In addition, the reproducibility of TE = 35 ms, 80 ms, and 144 ms was evaluated.

**Results:**

TE = 80 ms was the most reproducible for singlet metabolites with COVs < 6% for total N‐acetyl‐aspartate, total creatine, and total choline; however, moderate multiplet dephasing was observed. Analysis incorporating the experimental baseline achieved higher Glu and Glx reproducibility at TE = 35 ms, and showed improvements over the simulated baseline, with higher efficacy for poorer data.

**Conclusion:**

Overall, TE = 80 ms yielded the most reproducible singlet metabolite estimates. However, combined use of a short TE sequence and the experimental baseline may be preferred as a compromise between accuracy, multiplet dephasing, and T2 bias on metabolite estimates. Magn Reson Med 77:34–43, 2017. © 2016 The Authors Magnetic Resonance in Medicine published by Wiley Periodicals, Inc. on behalf of International Society for Magnetic Resonance in Medicine.

## INTRODUCTION

^1^H MR spectroscopic imaging (MRSI) has proven useful in the mapping of metabolite information across a volume of interest (VOI) 1, 2, 3. The identification of metabolite biomarkers has been shown to aid the diagnosis and prognosis of numerous diseases, such as brain tumors 4, 5, 6, neurodegenerative diseases 7, and neuro‐metabolic disorders 8, 9. Various factors can affect the accuracy to which MRSI data can be quantified, including hardware performance, data acquisition methods, and subsequent postprocessing techniques. Short‐echo MRSI metabolite quantitation accuracy can be reduced if macromolecular (MM) signals, originating from the resonances of cytosolic proteins and lipids 10, 11, are poorly characterized. These MM signals and any poorly defined baseline artefacts contribute to the background signal observed within MRSI spectra. These background signals can strongly overlap with metabolites of interest; therefore, any inaccuracies in MM estimations will decrease the accuracy of metabolite quantitation during the fitting process; this has been shown in several studies 12, 13, 14.

Several methods have been proposed to decrease the MM contribution or to characterize it for inclusion in the fitting process. One method that significantly reduces these background signals is to increase the echo time (TE) of the MRS acquisition, taking advantage of the shorter T2 relaxation times found for MM signals compared with metabolites 15 due to their smaller molecular weight 10, 16. However, at longer TEs, an inherent reduction in metabolite signal‐to‐noise ratio (SNR) due to T2 relaxation, and complex dephasing of multiplets can reduce quantitation accuracy. Therefore, short‐TE acquisitions may be better suited to measurement of multiplets and lower SNR metabolites.

The use of MM analysis schemes can help to characterize the MM contribution and account for it in the fitting process 16, improving the reliability of metabolite estimates. One widely used approach used is to include individual simulated macromolecule and lipid components within the analysis basis set 17. These features are typically derived from the parameterization of metabolite nulled spectra. Seeger et al parameterized the macromolecules by fitting an average MM spectrum to four broad lines, which included the main MM resonances 1–7; thus allowing prior knowledge to be introduced into the spectral fitting 17. MM baseline signals may also be experimentally acquired using an inversion recovery (IR) sequence 10 because metabolite signals can be nulled at inversion time (TI) due to shorter MM T1 relaxation. Small residual metabolite peaks are generally observed, but may be removed in post processing. A recent study by Snoussi et al 11 determined no significant differences between MM signals in white and gray matter, suggesting the use of a single MM baseline spectra could be sufficient to include in a basis set for the fitting of spectra from multiple brain regions.

MRS reproducibility studies are needed to assess reliability for the optimization of imaging protocols. Several of these have been reported previously; covering a large range of TEs, field strengths, and techniques 18, 19, 20, 21, 22, 23, 24. A recent study by Deelchand et al combined an experimental baseline with single voxel ^1^H MRS (SVS) for two sites within the brain and found highly reproducible neurochemical profiles could be achieved between scanners and regions providing optimized protocols were kept consistent 23. Previously comparisons have been made between the use of simulated baselines and experimental baselines in the fitting of spectra 12, 13, 25; however, their effect on the reproducibility of 2D ^1^H MRSI data has not previously been evaluated. In this study we investigate the effect of TE and two MM analysis schemes on the short‐term reproducibility of 1H MRSI data at 3 Tesla (T) to aid protocol design for clinical studies.

## METHODS

All MR scanning was performed on a 3T Philips Achieva TX MR system with a 32‐channel head coil at Birmingham Children's Hospital, UK.

### Human Subjects

All volunteers who participated in the study gave informed consent under full ethical approval.

### MRSI Data Collection

**Figure 1:**
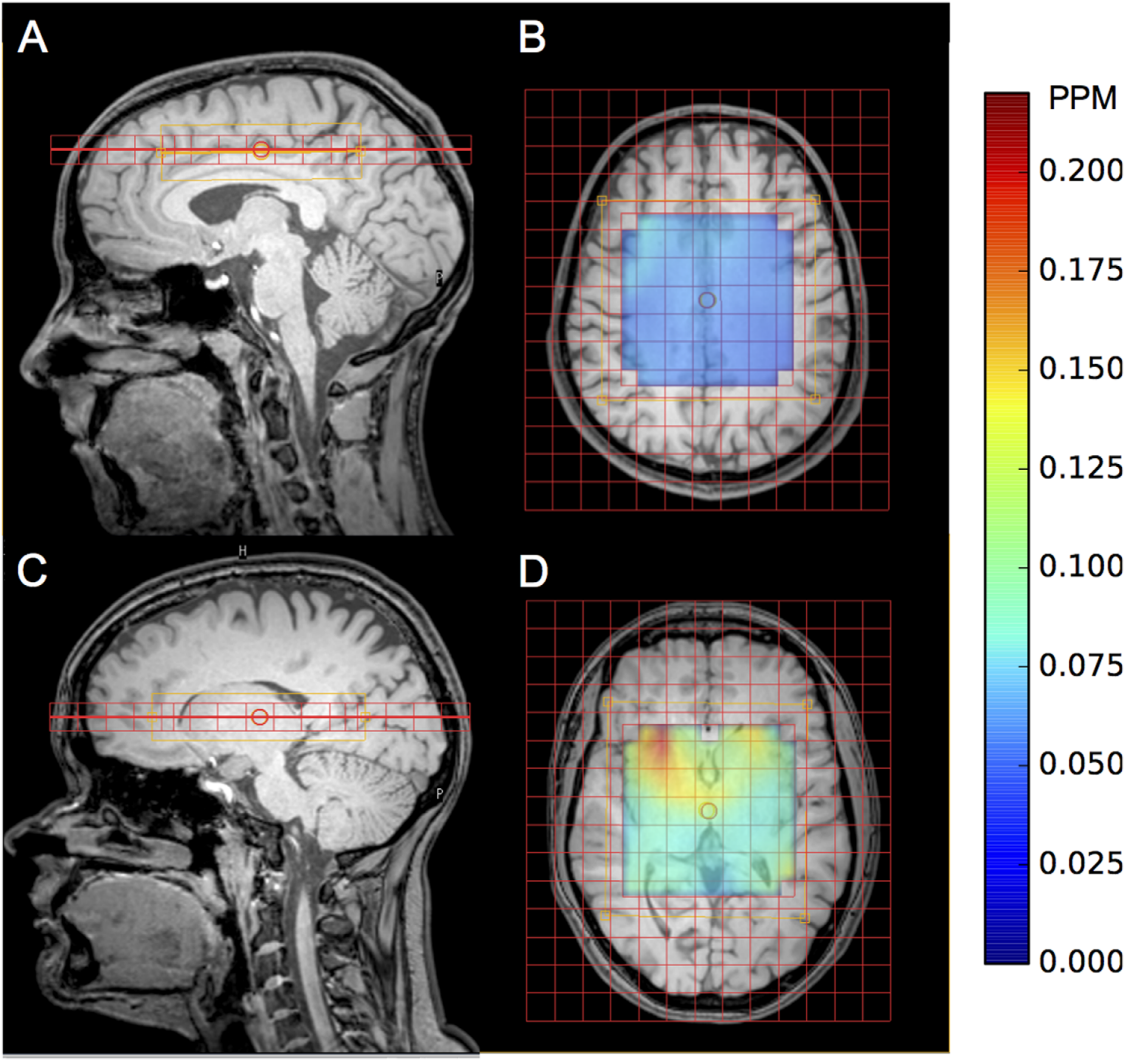
Example healthy volunteer MRSI grid placement for: above the corpus callosum sagittal (**A**) and axial (**B**) views and the basal ganglia sagittal (**C**) and axial (**D**) views. Water line width map is included for corpus callosum (B) and basal ganglia (D) for the PRESS excitation region.

All 2D MRSI data were collected from eight healthy volunteers (aged 23–30 years). An initial 3D T1 weighted anatomical MRI reference scan was obtained for MRSI grid positioning. All MRSI grids were manually placed for two regions: (A) above the corpus callosum (three volunteers), see Figures 1A and 1B; and (B) level with the basal ganglia (five volunteers), see Figures 1C and 1D, with the following acquisition parameters: field of view (FOV) matrix size 15 × 13 voxels; voxel size 13 mm × 13 mm × 13 mm; TR = 2 s. Pencil beam shimming was applied. Data were collected at three different TEs on separate volunteers: TE = 35 ms (region: A = 1 and B = 2 volunteers), TE = 80 ms (region: A = 1 and B = 1 volunteer), and TE = 144 ms (region: A = 1 and B = 2 volunteers). PRESS localization was used to excite a 6 × 6 voxel VOI (78 mm × 78 mm × 13 mm) fixed centrally to the FOV; corresponding to a fully excited 5 × 5 voxel region with a ½ voxel margin around the PRESS excitation region, edge voxels were discarded in post processing and the VOI extracted by means of a water signal threshold. To assess short‐term reproducibility, all scans were acquired in triplicate without grid repositioning; reshimming and water suppression frequency optimization in between scans was performed if deemed necessary. Due to lengthy scanning times, different volunteers were scanned for each TE and region. Absolute concentrations were obtained by collecting water unsuppressed data during the first scan for each volunteer using the same scanning parameters as the suppressed (metabolite) data collection.

### Spectral Fitting

All spectral analysis was performed using the TARQUIN software package 26. The TARQUIN algorithm performs a fully automated fit to the data using a predefined basis set containing metabolite, lipid and macromolecule signals. Fitting is performed in the time‐domain and constraints, such as nonnegative metabolite quantities, are enforced to improve reliability. The simulated MM analysis scheme (Si.BL) used the following basis set to fit the MRSI data comprised of 16 individually simulated components: alanine (Ala); aspartate (Asp); creatine (Cr) ‐CrCH2; gamma‐aminobutyric acid (GABA); glucose (Glc); glutamine (Gln); glutathione (Glth); glycerophosphorylcholine (GPC); myo‐inositol (Ins); lactate (Lac); lipid peaks at 0.9, 1.3a, 1.3b, and 2.0 ppm; macromolecules at 0.9, 1.2, 1.4, and 2.0 ppm; N‐acetyl‐aspartate (NAA); N‐acetyl‐aspartateglutamate (NAAG); phosphorylcholine (PCho); Phosphocreatine (PCr); scyllo‐inositol (s‐Ins); and taurine (Tau). All metabolite components were simulated in TARQUIN using chemical shift and J coupling values from Govindaraju et al 27. The simulated MM signal is taken to be the combination of the individual MM and lipid components listed in the paper describing the TARQUIN algorithm 26 (Table 1).

**Table 1:** Ex.BL and Its 13 mm Components Detailed According to Amplitude, Frequency, and FWHM

Name	Amplitude (AU)	Freq (PPM)	FWHM (Hz)
MM09	0.72	0.90	21.20
MM12	0.28	1.21	19.16
MM14	0.38	1.38	15.90
MM16	0.05	1.63	7.50
MM20	0.45	2.01	29.03
MM21	0.36	2.09	20.53
MM23	0.36	2.25	17.89
MM26	0.04	2.61	5.30
MM30	0.20	2.96	14.02
MM31	0.11	3.11	17.89
MM37	0.64	3.67	33.52
MM38	0.07	3.80	11.85
MM40	1.00	3.96	37.48

### Experimental Baseline Model Derivation (Ex.BL)

**Figure 2:**
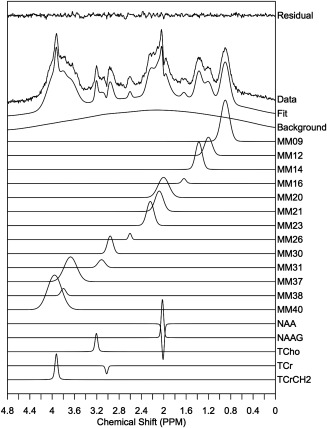
Average Ex.BL MM Spectra spectrum taken from seven healthy volunteers split into MM and residual metabolite components.

A single voxel spectroscopy (SVS) inversion recovery sequence was used to acquire metabolite nulled data for improved MM determination (TE/TR = 35/2500 ms PRESS excitation). An initial experiment was performed to establish the optimal inversion time for metabolite signal nulling. Spectra were acquired for TI = 500,650 to 850 ms in 50 ms steps and an optimal TI = 750 ms was determined. SVS inversion recovery data was collected from seven healthy volunteers (aged 23–30 years) from left and right parietal white matter regions. Ten voxels were chosen across the seven volunteers due to their high spectral quality, i.e., free from artefacts caused by spurious echoes and inefficient water suppression. These data were then compiled to create an average MM spectrum. The average spectrum was subsequently fitted using TARQUIN to obtain a noise free model of the MM baseline. A macromolecule basis set composed of Gaussian peaks was initially constructed based on known peak positions and visual inspection. This basis set was fit to the average spectrum allowing peak amplitudes, widths and frequencies to be optimized. The basis set was then updated with the newly determined frequencies and peak widths and this process of basis set refinement was repeated until fitting resulted in negligible adjustment to the basis set (Table 1). In addition to MM signals, narrow metabolite signals for NAA, NAAG, total choline (tCho), tCr, and tCrCH2 were also included in the basis set to account for metabolite signals that were partially nulled (Fig. 2). Finally, the optimized macromolecule peaks (excluding residual metabolite signals) were summed and added to a basis set of metabolite signals (described above), replacing the individual MM and lipid components. Analysis using this experimentally derived baseline model will be referred to as Ex.BL.

### Data Analysis

MRSI spectra exported from the scanner workstation in DICOM format were imported into TARQUIN. Spectra were fitted with two different basis sets: one including the Ex.BL and another that models the MM baseline using a set of individually simulated MM resonances (Si.BL). Subsequent data analysis was performed using Python programming language. Metabolite coefficients of variance (COVs) were calculated as the voxel wise ratio of the standard deviation and mean for each volunteer and TE over the three MRSI acquisitions. COVs were then grouped according to TE, irrespective of region, and a mean COV for each metabolite calculated. This was performed to avoid regional bias with respect to data quality. The VOI was extracted by means of a water amplitude threshold and only voxels with a water line width <0.1 ppm (12.8 Hz) were analyzed. Water line width was defined as the full width half maximum (FWHM) of the unsuppressed water peak in frequency domain. The unsuppressed water peak was taken to be out measure of data quality due to its robustness in comparison with metabolite line width, which can become unstable with noise and artifacts. We also assume that the water line width is directly correlated with metabolite FWHM and, therefore, can be used as a proxy for data quality. Metabolite COVs were calculated to assess technical reproducibility for TE = 35, 80, and 144 ms comparing the two MM analysis schemes: 1 Ex.BL and 2 Si.BL. COVs for the following metabolites were evaluated: total NAA (tNAA), total Cr (tCr), tCho, glutamate (Glu), glutamine (Gln), Glu + Gln (Glx), Tau, Glth, and Ins. An analysis of variance (ANOVA) test was performed for the metabolite COVs to test if a difference between the data sets from TE = 35, 80, and 144 ms were significant. Subsequent unpaired t‐tests were carried out to assess the statistical significance of any differences in reproducibility in comparison with TE = 80 ms, those with *P* < 0.05 were deemed significant.

Average white matter metabolite concentrations were also calculated to validate quantitation methodology. Metabolite concentrations were measured from white matter voxels selected using the axial T1 anatomical image registered to the MRSI grid. Average metabolite concentrations for 36 white matter voxels were taken from the slice going through the corpus callosum (12 from each scan) for TE = 35, 80, and 144 ms (3 volunteers total). This was done for both Si.Bl and Ex.Bl MM analysis schemes and a percentage difference in concentration was calculated between these two fitting methods. T2 relaxation correction was applied assuming a T2 of water of 60 ms and metabolite T2 of 200 ms.

## RESULTS

### Impact of TE and Ex.BL on Spectral Fitting

**Figure 3:**
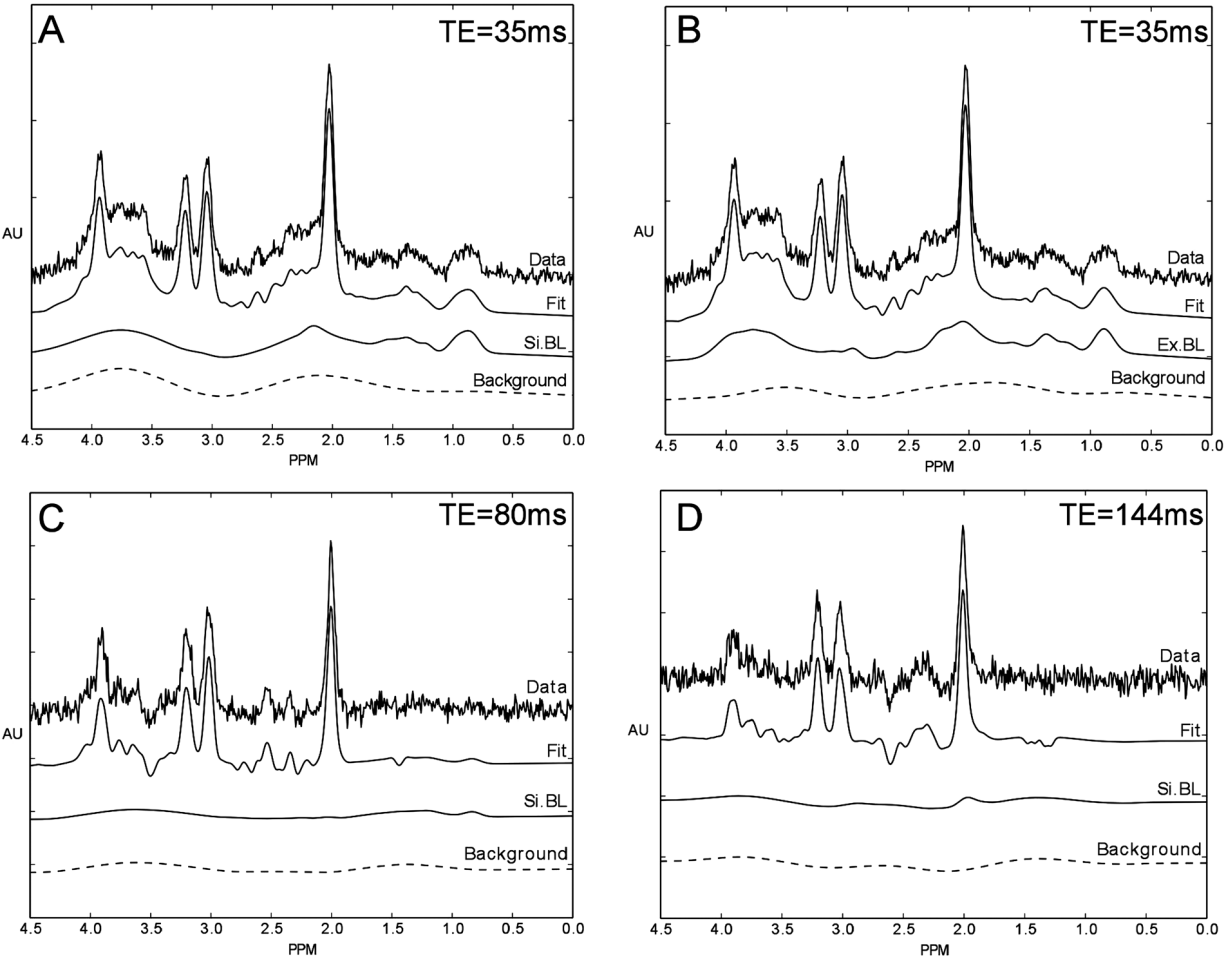
Example healthy volunteer spectra split into four components: data, fit, MM analysis scheme (Si.BL or Ex.BL) and residual background signal for three TEs: TE = 35 ms (Si.BL) (**A**); TE = 35 ms (Ex.BL) (**B**); TE = 80 ms (Si.BL) (**C**); and TE = 144 ms (Si.BL) (**D**).

The average MM spectrum across seven volunteers is shown in Figure 2 and the properties of the MM spectra decomposed into its 13 components are listed in Table 1. An example of the effect of the Ex.BL on the fitting of short TE spectra can be observed in Figures 3A and 3B. Broad background signals (obtained from a 50 data point moving average filter of the fit residual) are reduced with the inclusion of a better defined MM spectral content in comparison with a Si.BL. An increase in TE to 80 and 144 ms also reduced the level MM content relative to metabolites due to shorter T2 relaxation times (Figs. 3C, 3D). A reduction in SNR is also observed with increasing TE due to T2 relaxation and dephasing of metabolite multiplets.

### Effect of TE and MM Analysis Schemes on Reproducibility

Table 2 shows metabolite COV for voxels containing spectra that meet the water line width value criteria <0.1 ppm. Overall, COVs across the three TEs (35, 80, and 144 ms) were found to be statistically different by means of an ANOVA test for the following metabolites: tNAA, tCr, Glu, Gln, Ins, Glth, and Tau (*P* < 0.00001) for both the Si.BL and Ex.BL MM analysis scheme. Ex.BL Glx COV was also found to be statistically different (*P* = 0.0001). A TE = 80 ms was found to be the most reproducible with CoVs<9% for tNAA, tCho, and tCr. Additionally, TE = 80 ms showed improved reproducibility over TE = 35 ms (*P* < 0.01) for five of the metabolites analyzed: tNAA, tCr, Gln, Glth, and Tau for both MM analysis schemes (Table 2).

**Table 2:** Metabolite COVs for Voxels Containing Spectra That Meet Overall Water Line Width Criteria^a^

	0‐0.1 ppm							
	TE = 35ms		TE = 80ms		TE = 144ms		Echo time comparison	
Line width	COV %		COV %		COV %		ANOVA	
Metabolite	Si.BL	Ex.BL	Si.BL	Ex.BL	Si.BL	Ex.BL	Si.BL	Ex.BL
tNAA^††¥¥^	9.99	7.56	6.03	5.35	12.31	7.81	<0.00001	<0.00001
tCr	10.13	9.25	7.18	7.22	11.56	10.91	<0.00001	<0.00001
tCho	9.00	9.40	8.16	8.10	9.78	9.61	0.1209	0.1027
Glu^††^	20.35	16.33	22.36	22.82	45.96	47.57	<0.00001	<0.00001
Gln^¥^	83.80	88.85	56.22	60.19	47.37	53.22	<0.00001	<0.00001
Glx^††¥^	24.85	19.95	22.81	23.25	22.49	25.39	0.1525	0.0001
Ins	15.36	15.36	16.97	17.87	34.46	33.84	<0.00001	<0.00001
Glth^†^	48.43	68.14	28.33	27.67	69.06	68.39	<0.00001	<0.00001
Tau^††^	127.97	89.93	70.56	70.56	70.71	66.25	<0.00001	<0.00001

a Metabolite COVs for TE = 35, 80, and 144 ms for voxels containing spectra that meet the overall water line width criteria of between 0 and 0.1 ppm showing significant (*P* < 0.05) differences from an unpaired t‐test between Si.BL and Ex.BL for TE = 35 ms (†), TE = 80 ms (‡), and TE = 144 ms (¥). Significances of *P* < 0.01 are denoted as: TE = 35 ms (††), TE = 80 ms (‡‡), and TE = 144 ms (¥¥).

Across all TEs the most reproducible metabolites were found to be tNAA, tCr, and tCho with COVs < 13%. Higher variation at TE = 80 ms compared with TE = 35 ms was observed for Ex.BL Glu (*P* < 0.01) and Ex.BL Glx (*P* < 0.05) likely due to signal dephasing, therefore, a short echo acquisition maybe preferred depending on metabolite of interest. Long TE = 144 ms was found to have the highest variation between scans with Ex.BL COVs tNAA = 7.81%, tCr = 10.91%, and tCho = 9.61%; compared with 7.56%, 9.25%, and 9.4% for TE = 35 ms and 5.35%, 7.22%, and 8.1% for TE = 80 ms, respectively (Table 2). The use of an Ex.BL significantly reduces variation for TE = 35 ms compared with a Si.BL for tNAA, Glu, Glx, and Tau (*P* < 0.01). However, a Si.BL provided significantly better reproducibility for the measurement of Glth (*P* < 0.05).

### Effect of Line width and MM Analysis Schemes on Reproducibility

In addition to TE and analysis methodology, data quality is known to have a significant impact on the reproducibility of MRS data. Therefore, voxels were stratified into three line width groups: “good” 0–0.06 ppm (Table 3), “acceptable” 0.06–0.1 ppm (Table 4), and “poor” 0.1–0.15 ppm (Table 5) to investigate impact of line width on reproducibility. For tNAA, a ∼35% improvement in COV was observed between “good” (Table 3) and “acceptable” (Table 4) data using a Si.BL analysis, whereas a smaller improvement of ∼22% was found for the Ex.BL analysis.

**Table 3:** Metabolite COVs for Voxels Containing Spectra That Meet “Good” Water Line Width Criteria^a^

	0‐0.06 ppm							
	TE = 35 ms		TE = 80 ms		TE = 144 ms		Echo time comparison	
Line width	COV %		COV %		COV %		ANOVA	
Metabolite	Si.BL	Ex.BL	Si.BL	Ex.BL	Si.BL	Ex.BL	Si.BL	Ex.BL
tNAA¥¥	7.79	6.59	4.07	3.81	10.96	7.55	<0.00001	<0.00001
tCr†	10.52	8.69	4.70	4.25	10.19	9.90	<0.00001	<0.00001
tCho	8.16	8.19	5.91	5.75	9.16	8.81	<0.05	<0.05
Glu††	17.30	13.73	16.80	17.87	37.08	41.52	<0.00001	<0.00001
Gln	83.98	93.24	41.61	46.79	46.91	52.31	<0.00001	<0.00001
Glx††	19.54	15.39	18.55	19.80	22.01	24.50	<0.05	<0.00001
Ins	15.94	15.69	10.24	10.94	27.00	27.22	<0.00001	<0.00001
Glth	33.08	38.41	27.34	25.37	62.60	61.22	<0.00001	<0.00001
Tau††	127.87	89.93	62.30	60.61	60.63	56.44	<0.00001	<0.00001

a Metabolite COVs for TE = 35, 80, and 144 ms for voxels containing spectra that meet the “good” water line width criteria of between 0 and 0.06 ppm showing significant (*P* < 0.05) differences from an unpaired t‐test between Si.BL and Ex.BL for TE = 35 ms (†), TE = 80 ms (‡), and TE = 144 ms (¥). Significances of *P* < 0.01 are denoted as: TE = 35 ms (††), TE = 80 ms (‡‡), and TE = 144 ms (¥¥).

**Table 4:** Metabolite COVs for Voxels Containing Spectra That Meet “Acceptable” Water Line Width Criteria

	0.06‐0.1ppm							
	TE = 35 ms		TE = 80 ms		TE = 144 ms		Echo time comparison	
Line width	COV %		COV %		COV %		ANOVA	
Metabolite	Si.BL	Ex.BL	Si.BL	Ex.BL	Si.BL	Ex.BL	Si.BL	Ex.BL
tNAA††¥¥	11.94	8.41	6.77	5.92	14.73	8.27	<0.00001	<0.0001
tCr	9.78	9.74	8.12	8.33	14.02	12.73	<0.00001	<0.001
tCho	9.75	10.47	9.00	8.98	10.89	11.03	0.2815	0.1056
Glu†	23.06	18.63	24.45	24.68	62.45	59.21	<0.00001	<0.00001
Gln	83.65	85.64	61.78	65.36	48.18	54.81	<0.00001	<0.00001
Glx†	29.56	23.99	24.41	24.54	23.34	26.99	0.01	0.3328
Ins	14.85	15.06	19.49	20.47	47.85	45.72	<0.00001	<0.00001
Glth†	63.00	102.50	28.71	28.53	92.26	88.03	<0.00001	<0.00001
Tau††	128.16	96.08	73.74	74.43	91.93	86.37	<0.00001	<0.01

Metabolite COVs for TE = 35, 80, and 144 ms for voxels containing spectra that meet the “acceptable” water line width criteria of between 0.06 and 0.1ppm showing significant (*P* < 0.05) differences from an unpaired t‐test between Si.BL and Ex.BL for TE = 35 ms (†), TE = 80 ms (‡), and TE = 144 ms (¥). Significances of *P* < 0.01 are denoted as: TE = 35 ms (††), TE = 80 ms (‡‡), and TE = 144 ms (¥¥).

**Table 5:** Metabolite COVs for Voxels Containing Spectra That Meet “Poor” Water Line Width Criteria^a^

	0.1‐0.15 ppm			
	TE = 35 ms		TE = 80 ms	
Line width	COV %		COV %	
Metabolite	Si.BL	Ex.BL	Si.BL	Ex.BL
tNAA††‡‡	23.09	14.39	12.96	6.53
tCr	15.53	14.92	10.21	10.22
tCho	15.15	14.17	18.44	17.44
Glu	30.62	27.46	44.72	40.21
Gln	56.13	56.46	92.37	101.36
Glx	23.99	23.75	42.56	38.28
Ins	27.85	27.22	34.79	38.10
Glth†	103.15	121.31	44.15	46.19
Tau	141.42	131.06	73.90	77.15

a Metabolite COVs for TE = 35 and 80 ms for voxels containing spectra that meet the “poor” water line width criteria of between 0.1 and 0.15 ppm showing significant (*P* < 0.05) differences from an unpaired t‐test between Si.BL and Ex.BL for TE = 35 ms (†), TE = 80 ms (‡), and TE = 144 ms (¥). Significances of *P* < 0.01 are denoted as: TE = 35 ms (††), TE = 80 ms (‡‡), and TE = 144 ms (¥¥).

Improved reproducibility for the Ex.BL analysis was found for poorer water line widths when compared with Si.BL analysis. The Ex.BL analysis resulted in a large reduction of 38% in the COV of tNAA for line widths between 0.1 and 0.15 ppm (Table 5). Smaller reductions of ∼30% and ∼15% were found for line widths between 0.06–0.1ppm and 0.0–0.06ppm, respectively. Improved agreement in reproducibility between the two analysis methods was found with increasing TE for tNAA, tCr, tCho, Glx, and Tau (Tables 3, 4), likely due to the reduction in interference from MM signals at longer TEs.

The effect of imaging region on water line width can be observed in Figures 1B and 1D; with a line width approximately three times higher for the basal ganglia imaging slice compared with a higher slice above the corpus callosum. Across both regions, a mean water line width of 0.07, 0.07, and 0.06 ppm were observed for TE = 35, 80, and 144 ms, respectively for voxels analyzed with the water line width criteria of <0.1 ppm. Poor line widths were particularly observed in the frontal region of the basal ganglia slice through the brain. Magnetic field inhomogeneity is commonly observed in MRSI data when there is a close proximity to air‐tissue interfaces 28 such as the sinuses, resulting in spectral line broadening. Poor line width increased fitting error resulting in a reduction in overall reproducibility. For a TE of 35 ms, 38% of the total voxels across all scans and volunteers were discarded due to poor line width (>0.1 ppm); compared with 14% and 3% for TE = 80 ms and 144 ms, respectively. Analysis showed that the location of these voxels for the short echo data were predominantly in the basal ganglia region.

### Effect of TE and MM Analysis Schemes on Metabolite Quantitation

Average white matter metabolite concentrations were also calculated to validate quantitation methodology. Table 6 shows the absolute concentrations for the metabolites taken from white matter for each TE and a comparison between Si.BL and Ex.BL analysis schemes. For TEs = 35 and 80 ms, absolute concentrations were found to be consistent with those found in literature for healthy parietal white matter 19, 29. However, a slight underestimation was observed for TE = 144 ms suggesting accuracy may be reduced at long TEs due to increased T2 relaxation. It is also worth noting that differences in metabolite concentrations across TE could be attributed to natural variability across volunteers. The percentage difference in concentration between Si.BL and Ex.BL MM analysis schemes was found to be below 23% across all TEs for tNAA, tCr, and tCho. Larger differences were observed for poorly determined metabolites such as Gln, Glth, and Tau, particularly at TE = 35 ms.

**Table 6:** Average Healthy Volunteer Metabolite Concentrations for Left and Right White Matter^a^

	TE = 35 ms			TE = 80 ms			TE = 144 ms		
Metabolite	Mean concentration (mmol)		Δ (%)	Mean concentration (mmol)		Δ (%)	Mean concentration (mmol)		Δ (%)
	Si.BL	Ex.BL		Si.BL	Ex.BL		Si.BL	Ex.BL	
tNAA	7.0	6.5	7.2	7.6	7.8	−1.3	5.7	6.6	−14.7
tCr	4.9	4.5	9.3	3.6	3.5	1.4	2.3	2.3	−0.1
tCho	1.4	1.3	9.1	1.1	1.1	1.6	1.0	1.0	3.6
Glu	5.2	5.3	−2.9	4.0	4.0	0.0	1.0	0.9	17.2
Gln	0.8	0.5	45.3	0.5	0.5	10.2	1.2	1.0	17.5
Glx	6.0	5.8	3.9	4.5	4.5	1.2	2.2	1.8	17.4
Ins	2.8	2.9	−3.5	3.6	3.5	1.8	1.3	1.3	−0.3
Glth	1.0	0.3	66.1	0.7	0.8	−1.1	0.2	0.2	−34.5
Tau	0.1	0.5	−543.6	0.4	0.4	−1.8	0.4	0.4	−2.9

a Average healthy volunteer metabolite concentrations for left and right white matter for TE = 35, 80, and 144 ms for both Ex.BL and Si.BL MM analysis schemes. A percentage difference Δ between Si.BL and Ex.BL is also shown.

## DISCUSSION

The effect of TE and MM analysis schemes on the reproducibility of ^1^H MRSI has been evaluated for three different TEs (35, 80, and 144 ms) and a comparison made between two different MM analysis schemes Si.BL and Ex.BL. Overall, a TE = 80 ms was found to be significantly more reproducible for five metabolites: tNAA, tCr, Gln, Glth, and Tau. For the determination of Glu and Glx, however, a TE = 35 ms in conjunction with Ex.BL performed significantly better.

As expected, the most reproducible metabolites were found to be tNAA, tCr, and tCho due to their higher SNR resulting in an increase in their fitting precision. The improved reproducibility with the use of an Ex.BL compared with a Si.BL in the fitting of short echo spectra was found to be significant for four metabolites: tNAA, Glu, Glx, and Tau. In addition, increased improvements were observed for spectra with poorer line widths, suggesting the Ex.BL method could be used to improve the reliability of poor quality data.

A recent study by Veenith et al 30 found within session reproducibility COVs of 9.9% for tNAA, 10.6% tCr, and 14.3% for tCho at TE = 70 ms compared with our values of 6.03%, 7.18%, and 8.16% for tNAA, tCr, and tCho, respectively, using a simulated MM baseline at TE = 80 ms. A slight improvement was observed for TE = 80 ms with the use of an Ex.BL for these metabolites with COVs of 5.35% and 8.1% for tNAA and tCho, respectively, within our study. Improved reproducibility found within our study at intermediate TE could be attributed to several differences in methodology, some of which include: SPIN‐ECHO versus PRESS acquisition, the use different analysis software and a slightly different line width threshold of <12 Hz. Our protocol also had a slightly greater TE, allowing further decay of MM signals, potentially reducing the influence of these signals on the overall reproducibility.

Another study by Gasparovic et al 31 at 3T used a short TE of 40 ms and found comparable COVs of 5% for tNAA, 6% Cr, 7% Cho, 11% Ins, 10% Glu, and 13% for Glx. Their TE = 40 ms was chosen for optimized detection of Glu. Additionally, a study by Jang et al 32 at 1.5T also found an optimal short TE of 40 ms using PRESS for the measurement of Glu with comparable COVs of 11 and 13.1% for anterior cingulate cortex and insula, respectively. In our study at TE = 35 ms, a COV of 16.33% was found for Glu which may be because our sequence has not been specifically optimized for Glu.

There is growing interest in MRS measures of Glu for the study of schizophrenia 33; brain tumor metabolism 34, and cognition 35. In this study, the combination of Ex.BL and a 35 ms TE yielded the most reproducible measure of Glu (COV = 14%). This finding is in agreement with a previous study by Hancu, where simulations and in vivo data were used to demonstrate that shorter TE acquisitions showed better reproducibility for Glu measurements 36. Work by Mullins et al also found a shorter TE to be optimal for Glu with COVs of 2.6% for TE = 40 ms and 7.6% for TE = 80 ms 37. Contrary to this, a study by Schubert et al 38, assessed the determination of Glu using SVS at 3T, proposed an intermediate TE of 80 ms to be optimal. However, Schubert et al focused on TEs between 50 ms and 330 ms and did not evaluate short‐echo spectra < 50 ms.

In addition, MM signals were not included in the analysis basis set, therefore, analysis of TEs shorter than 50 ms would have resulted in greater interference from MM signals. At TE = 80 ms, a COV of 17% for Glu was achieved in this study compared with 10% from Schubert et al, this difference may be attributed to the generally higher data quality available from SVS. Although this study was focused on MRSI, it is worth noting that the findings here broadly agree with similar SVS studies measuring Glu. This indicates our results may also be applicable to the design of SVS studies. Hancu and Port et al 39 suggest a TE = 80 ms is optimal for Gln with in vivo COVs of 12.7% compared with 122% for a TE = 35 ms. Due to dephasing a shorter TE was better for Glu with simulation COVs of 4.2% for TE = 35 ms and 5.1% for TE = 80 ms. This finding is in agreement with our study. Our study, however, shows increased variation for Glu as expected in vivo when compared with simulation. J‐modulation effects for Glu at longer TE could explain why Glu is dephased at longer TE, whereas Gln can still be reliably measured. It is also worth noting that as Gln is present in low levels its reproducibility can be difficult to determine accurately.

Increased MRS measures of Lac are thought to be associated with hypoxia or ischemia; therefore, this signal is widely measured in clinical MRS studied to investigate pathologies such as brain tumors and mitochondrial disease 40. A TE of 144 ms is often used for the measurement of Lac, due to a characteristic signal inversion at this TE and improved separation for lipid signals at 1.3 ppm 40, 41. Due to the low levels of Lac in normal brain, this metabolite was not considered in this study; however, this example highlights that a single TE may not be optimal for all metabolites. Therefore, protocol optimization will always benefit from prior knowledge of the important metabolites for a particular clinical question. However, in the absence of a prior hypothesis, a shorter TE with appropriate MM and lipid analysis will give good results for most metabolites due to reduced multiplet dephasing and T2 weighting.

It is worth noting that, in addition to metabolites, MM spectral information is also of clinical interest, since it has been shown to provide useful information for several diseases, such as brain tumors where MM content was found to be useful in assigning tumor grade 42. A recent study by Craveiro et al has shown that mouse models of human glioma have exhibited several alterations in the macromolecule spectrum when compared with healthy controls corresponding to mobile lipids and also a broad MM component between 3.6 and 3.7 ppm. This introduced significant errors into the quantification of Lac and Asp 43. Therefore, any difference in MM content due to pathology may need to be accounted for to maintain accurate metabolite quantification. This also suggests that a MM fitting model based on normal tissue may not be suitable for the analysis of some pathology. Elevated levels of MM have also been observed in studies of multiple sclerosis 44 and stroke 45, 46. However, the acquisition of MM spectra using an inversion recovery sequence for each patient would prove time consuming and may preclude its use for routine clinical use. Therefore, because pathology MM content is likely to differ from normative profiles in some diseases, the more flexible Si.BL analysis method should be chosen in the absence of a patient or disease specific MM profile.

Whereas both TE and MM analysis schemes can influence the reproducibility of ^1^H MRSI spectra, data quality parameters such as water line width and SNR also have a significant impact. TE is inherently linked with SNR; therefore, it is an important factor to consider for protocol optimization. Loss of signal limits the amount of metabolite information that can be observed at increased TEs, as lower level signals get closer to background levels decreasing fitting accuracy. Water line width is another issue that can influence the reproducibility of spectra. It can be observed from Table 5 that, if a rejection criterion is not set, the available reproducibility for each metabolite can increase drastically. For example, a twofold change in reproducibility can be observed in the case of tNAA compared with water line width <0.01 ppm. Water line width is also dependent on imaging location, meaning voxel placement may become an issue in protocol design. In this study, poorer water line widths were observed for spectra collected with a slice going through the basal ganglia, particularly in the frontal region, due a closer proximity to susceptibility inducing air–tissue boundaries, e.g., sinuses. This proved particularly problematic for short‐echo spectra, resulting in the rejection of over a third of voxels due to water line width (>0.01 ppm). This loss of information would prove particularly disadvantageous if the voxels discarded are located in an area of pathological interest. Although an apparent relationship between TE and water line width was observed in our data, we regard this as a chance observation arising from random subject variability, rather than a causal relationship.

This study demonstrates several factors to be considered in MRSI protocol optimization including: TE, MM analysis schemes, metabolite of interest, location and data quality, all which are not necessarily independent from each other. This study mainly focuses on the intrasubject reproducibility within the same session rather than accuracy. Metabolite concentrations were calculated for each TE and MM analysis scheme and were found to be consistent with those previously published in literature for parietal white matter 19, 29. A slight underestimation was found for a TE = 144 ms, suggesting accuracy is decreased at longer TE, which could be due to T2 bias on the metabolite estimates. True measures of accuracy are difficult to establish in volunteers and patients due to a lack of alternative methods for measuring NMR visible metabolite concentrations. For this reason, partial volume corrections for white matter, gray matter, and cerebrospinal fluid were not required for this study, as COV measured were only performed between voxels with identical tissue constituents. Further work is needed to develop methods for establishing combined reproducibility and accuracy.

## CONCLUSIONS

In this study, TE, MM baseline analysis, and data quality were all found to impact the reproducibility of ^1^H MRSI data. These results highlight the importance of careful selection of TE and analysis methodology where optimal reproducibility is sought for a primary metabolite of interest. Overall, this study found that a TE = 80 ms produced the most reproducible metabolite values for tNAA, tCho, tCr, and Glth in healthy volunteers. However, the combined use of a short TE sequence and the MM analysis scheme Ex.BL may be preferred as a compromise between good accuracy, good SNR and T2 bias on metabolite estimates.
